# Stabilizing superconductivity of ternary metal pentahydride $\hbox {CaCH}_{{5}}$ via electronic topological transitions under high pressure from first principles evolutionary algorithm

**DOI:** 10.1038/s41598-022-10249-1

**Published:** 2022-04-25

**Authors:** Prutthipong Tsuppayakorn-aek, Nakorn Phaisangittisakul, Rajeev Ahuja, Thiti Bovornratanaraks

**Affiliations:** 1 Extreme Condition Physics Research Laboratory and Center of Excellence in Physics of Energy Materials (CE:PEM), Department of Physics, Faculty of Science, Chulalongkorn University, Bangkok, 10330 Thailand; 2 Thailand Centre of Excellence in Physics, Ministry of Higher Education, Science, Research and Innovation, 328 Si Ayutthaya Road, Bangkok, 10400 Thailand; 3 Condensed Matter Theory Group, Department of Physics and Astronomy, Uppsala University, Box 530, SE-751 21 Uppsala, Sweden; 4 Department of Physics, Indian Institute of Technology (IIT) Ropar, Rupnagar, 140001 Punjab India

**Keywords:** Condensed-matter physics, Theory and computation

## Abstract

We explored the phase stability of ternary pentahydride $\hbox {CaCH}_{{5}}$ based on the first principles evolutionary algorithm. Here, we successfully search for a candidate structure up to 500 GPa. As a consequence, the possible stable structure of $\hbox {CaCH}_{{5}}$ is found be to a monoclinic structure with space group *Pm* at a pressure of 50 GPa. Moreover, the orthorhombic structure with a space group of *Cmcm* is found to be thermodynamically stable above 316 GPa. With this, the Kohn-Sham equation plays a crucial role in determining the structural stability and the electronic structure. Therefore, its structural stability is discussed in term of electronic band structure, Fermi surface topology, and dynamic stability. With these results, we propose that the superconducting transition temperature ($\hbox {T}_{{c}}$) of *Cmcm* structure is estimated to be 50 K at 450 GPa. This could be implied that the proposed *Cmcm* structure may be emerging as a new class of superconductive ternary metal pentahydride. Our findings pave the way for further studies on an experimental observation that can be synthesized at high pressure.

## Introduction

Hydrogen (H) was proposed for high-temperature superconductivity by Ashcroft^1^, Its outstanding property suggested that the high-temperature superconductor considered the Bardeen–Cooper–Schrieffer (BCS) theory^2^, namely, the high-temperature superconductor can be predicted from the high Debye temperature, as revealed by the BCS. About 30 years later, theoretical works^3,4^ pointed out the ability of hydrides as high-temperature superconductivity when hydrogen is a dominant ingredient. For example, the superconducting transition temperature ($\hbox {T}_{{c}}$) of $\hbox {H}_{{3}}$S and $\hbox {LaH}_{{10}}$ were reported at 203 K and 250 K, respectively^5,6^, besides, several metal hydrides have been proposed to be conventional superconductors^7–18^. These materials successfully demonstrated the importance of metallic hydrogen, showing that they play an important role in the enhancement of superconductivity.

Recently, the ternary Ca–B–H system was investigated by the first-principles calculations. Cataldo et al.^19^ owed their structures are metallization, leading to superconductivity. Two compositions of this system, i.e. $\hbox {CaBH}_{{6}}$ and $\hbox {Ca}_{{2}}\hbox {B}_{{2}}\hbox {H}_{{13}}$, were predicted to be high-temperature superconductor. Additionally, the $\hbox {T}_{{c}}$ value of $\hbox {CaBH}_{{6}}$ is an exclusive superconductor with a $\hbox {T}_{{c}}$ of 119 K at 300 GPa and $\hbox {Ca}_{{2}}\hbox {B}_{{2}}\hbox {H}_{{13}}$ is a superconductor with a $\hbox {T}_{{c}}$ of 8 K at a pressure of 300 GPa. Meanwhile, theoretical studies^19^ revealed that the $\hbox {T}_{{c}}$ value of $\hbox {CaBH}_{{5}}$ is 0.1 K at a pressure of 300 GPa. Following this, $\hbox {CaBH}_{{5}}$ was shown despite being metallic but for the fact that it is nonsuperconducting due to a poor phonon coupling between boron and hydrogen^19^. Recent extensive studies in $\hbox {H}_{{3}}$S, the literature reports attempts to explore higher transition temperatures by studying hole-doped $\hbox {H}_{{3}}$S system such as $\hbox {S}_{1-x}\hbox {P}_{{x}}\hbox {H}_{{3}}$, $\hbox {S}_{1-x}\hbox {Si}_{{x}}\hbox {H}_{{3}}$, and $\hbox {S}_{1-x}\hbox {C}_{{x}}\hbox {H}_{{3}}$, respectively^17,20^. These system pave the way to stabilizing ternary sulfur trihydride structures at high pressures. Following this, it should be noted that the C-S-H system has been widely interested in superconductor research because Ge et al.^20^ pointed out that carbonaceous sulfur hydride exhibited a high-temperature superconductivity with 289 K at a pressure of 260 GPa.

As mentioned Ca-B-H system, particularly in $\hbox {CaBH}_{{5}}$, it is interesting to note that couple between B and H phonons is poor. As a result of this, $\hbox {CaBH}_{{5}}$ displayed lower transition temperatures. Therefore, it is interesting to explore another possibility for ternary metal pentahydride ($\hbox {TMH}_{{5}}$). Meanwhile, it is worth noting that the existence of carbon in ternary sulfur trihydride shown that its $\hbox {T}_{{c}}$ (289 K) is higher that $\hbox {H}_{{3}}$S (203 K). As a consequence, carbon substitution is occurred in boron site because of the similarity between boron and carbon in the atomic characteristics. Also, it is interesting to investigate the fundamental physics of an unknown compound at high pressure. Keeping all these recent motivations in mind; there are several open questions for $\hbox {CaCH}_{{5}}$ under compression: (i) What is a new structure of $\hbox {CaCH}_{{5}}$ at high pressure? (ii) What is a physical property in a candidate structure? (iii) Does the metallic high-pressure phase become a superconductor?

In this work, we predict a novel structure of $\hbox {CaCH}_{{5}}$, leading to scientific issues of high pressure. We explore the high-pressure phase of $\hbox {CaCH}_{{5}}$ under high pressure from 50 GPa to 500 GPa, by the first-principles evolutionary techniques. Additionally, our calculation suggested that a ternary superconducting phase of $\hbox {CaCH}_{{5}}$ is thermodynamically stable up to at least 500 GPa and is going to be further discussed in the result and discussion. To further understand the superconductivity of $\hbox {CaCH}_{{5}}$, it is interesting to note that role of Pauling electronegativity was also theoretically investigated by Xie et al.^16^. Herein, it has been reported in the literature that the elements of Hf and Zr is similar Pauling electronegativity and they are carried out with hydride, leading to high-T_c_ superconductivity. Following this, it might be worth trying to adopt a similar Pauling electronegativity in carbon for consideration of high-$\hbox {T}_{{c}}$ superconductivity of $\hbox {CaCH}_{{5}}$ because the Pauling electronegativity of carbon is similar the Pauling electronegativity of boron. According to aforementioned theoretical study findings^19^. These findings provide crucial details for fundamental understanding of the phase diagram and the electronic properties of of Ca–C–H system at high pressure.

We are now focusing on the $\hbox {CaCH}_{{5}}$ because it displayed a significant issue for ternary pentahydride. This is, however, beyond the scope of phase diagram of Ca–C–H system, and we point out that the issue should clearly deserve further investigation for consideration a phase diagram of Ca–C–H system. In brief, a novel structure started to examine the ground state structure and in comparison to $\hbox {CaBH}_{{5}}$ at high pressure. Moreover, we study electronic property as well as electronic topological transition, which is an electronic band structure up to 300 GPa, leading to a stabilizing superconductivity of $\hbox {CaCH}_{{5}}$ above 300 GPa.

## Methods

High pressure structures of calcium carbohydrides $\hbox {CaCH}_{{5}}$ were calculated by first-principles evolutionary techniques, as implemented the Universal Structure Predictor: Evolutionary Xtallography (USPEX)^21^. In all subsequent generations, the random symmetric algorithm employed 40$\%$ heredity, 20$\%$ random symmetric, 20$\%$ soft mutation, and 20$\%$ transmutation operators. We explored systems in the pressure range from 50 to 500 GPa with up to four formula units. The 2979 configurations were considered by the lowest enthalpy in 25 consecutive generations. All structures were fully relaxed using the generalized gradient approximation of the Perdew–Burke–Ernzerhof (GGA-PBE) functional^22^ for the exchange-correlation functional. We performed the projector augmented wave (PAW) method^23^ and the conjugate gradient scheme, as implemented in the Vienna ab initio simulation package (VASP)^24^. The pseudocore radii of Ca, C, and H are 2.3 Bohr, 1.1 Bohr, and 0.8 Bohr, which are small enough to ensure no overlap of spheres will occur under compression. A plane-wave basis set up to cutoff energy of 700 eV and an initial Brillouin-zone (BZ) sampling grid of spacing 2$\pi$ $\times$0.02 Å$^{-1}$ were used for this calculation. The dynamic stable structures were calculated by using the *ab initio* lattice dynamics, as implemented in the VASP code combined with the PHONOPY package^25^. For electron-phonon calculations, a plane-wave energy cutoff of 60 Ry was used. The Eliashberg spectral function and electron phonon coupling (EPC) with density functional perturbation theory^26^ were calculated using the Quantum espresso (QE) code^27^. The EPC matrix elements were computed in the first BZ on 4$\times$4$\times$2 q-meshes using individual EPC matrices obtained with 24$\times$24$\times$16 k–points mesh. The Allen–Dynes equation^28^ was used with the effective Coulomb pseudopotential parameter, $\mu ^{*}$= 0.10. as follows:

1 $$\begin{aligned} T_{c} = \frac{\omega _{log}}{1.2} \exp \Big [ -\frac{1.04(1+\lambda )}{\lambda -\mu ^*(1+0.62\lambda )} \Big ], \end{aligned}$$

where $\omega _{log}$ is the logarithmic average of the spectral function. $\lambda$ is the total electron-phonon coupling strength.

## Results and discussion

**Figure 1:**
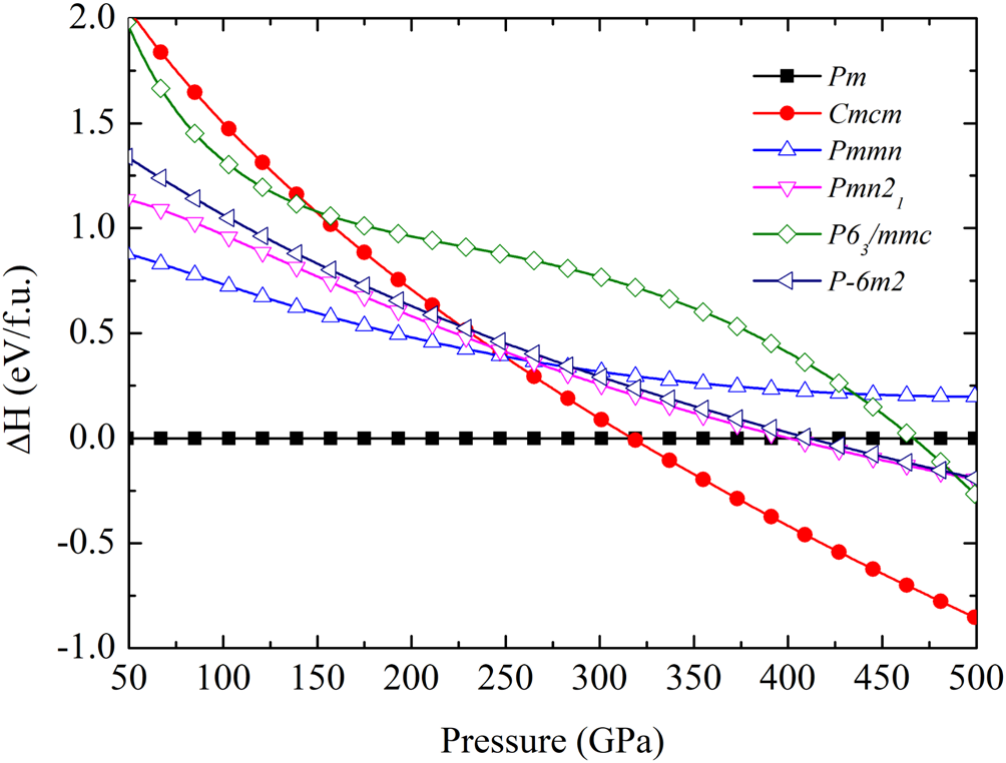
The relative enthalpy as a function of pressure ranging from 50 GPa to 500 GPa of $\hbox {CaCH}_{{5}}$.

**Figure 2:**
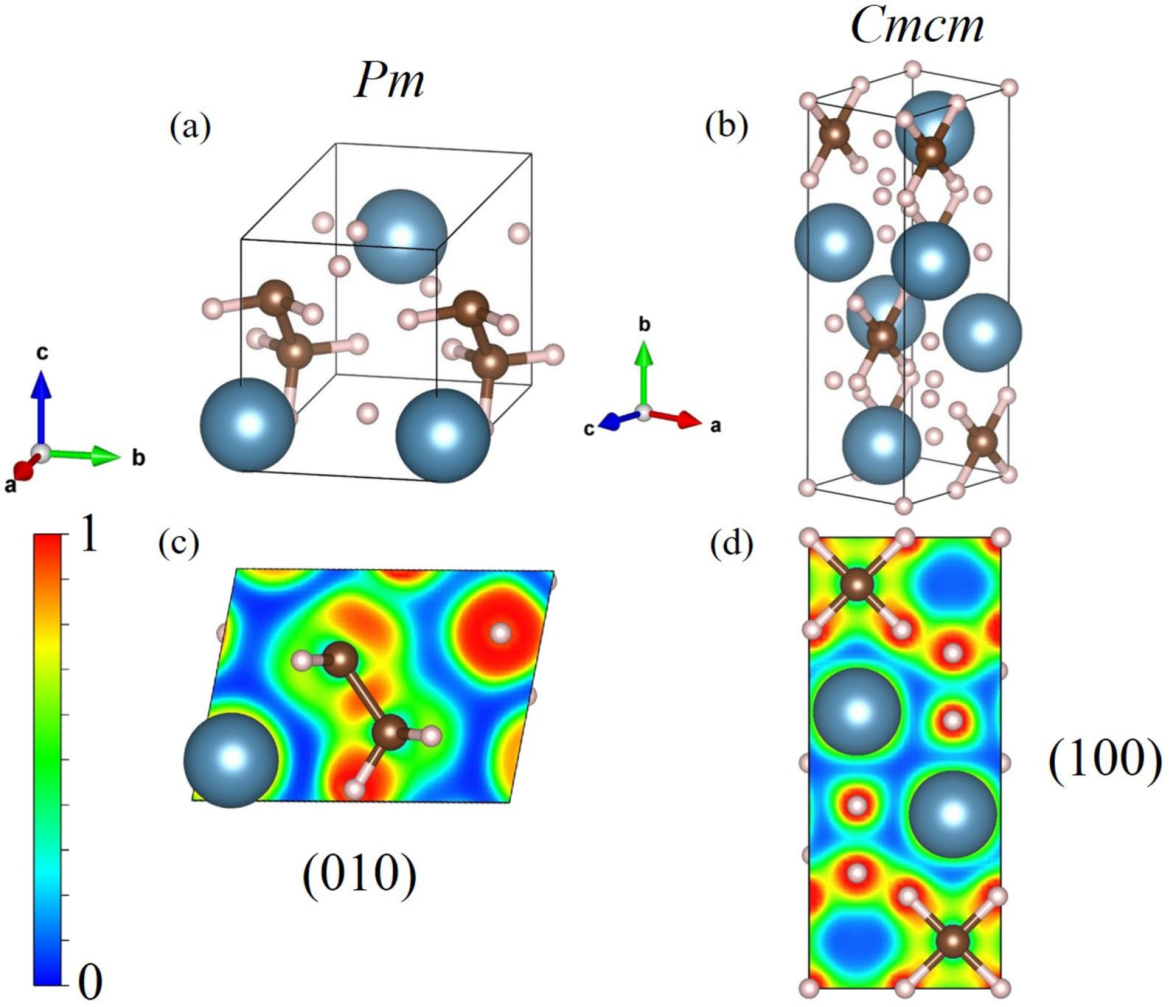
A schematic illustration of the *Pm* structure and the *Cmcm* structure. The Ca atoms are shown in dark blue, the C atoms in brown, and the H atoms in pink colour (drawn by VESTA (ver. 3.4.7)^56^ (URL https://jp-minerals.org/vesta/en/download.html)).

Regarding the ground-state energy of $\hbox {CaCH}_{{5}}$, the high-pressure phase was predicted by USPEX code. To search for the stable structure, the possible stable structure with respect to a monoclinic structure with a space group of *Pm* were calculated. Our main structural prediction results showed that the *Pm* structure is thermodynamically stable at a pressure of 50 GPa. On further compression to 500 GPa, it was then transformed into an orthorhombic structure with a space group of *Cmcm* at a pressure of 316 GPa (Fig. 1). As a result of this, the enthalpy of *Cmcm* structure is declined steadily up to 500 GPa. Along with, we found that the *Pm* structure is thermodynamically stable at a pressure of 200 GPa, and the *Cmcm* is more thermodynamically stable favored over the *Pm* structure between 400–500 GPa. Besides, we confirmed the stability of $\hbox {CaCH}_{{5}}$ against the phase decomposition into the CaC and H phases up to at least 500 GPa, With this, we consider the formation enthalpy of $\hbox {CaCH}_{{5}}$ respect to CaC^29^ and H^30^, depicting in the convex hull envelopes of Fig. S1 in the Supplemental Material. The detail of morphology and structural parameter showed in Figs. 2a, 2b, and Table 1, respectively. This implied that $\hbox {CaCH}_{{5}}$ can be synthesized through the formation of CaC and H. Moreover, the ground state structure of $\hbox {CaCH}_{{5}}$ will be confirmed by considering the phonon calculation, as discussed later.

**Table 1:** Structures of $\hbox {CaCH}_{{5}}$

Space group	Pressure (GPa)	Lattice parameter Å, ($^{\circ }$)	Atomic coordinate (fractional)
*Pm*	50	a = 5.116 b = 3.444 c = 3.952	Ca1 (0.9308, 0.0000, 0.16790)
		$\alpha$ = 90 $\beta$ = 99.32 $\gamma$ =120	Ca2 (0.3545, 0.5000, 0.76834)
			C1 (0.4377, 0.0000, 0.2844)
			C2 (0.6398, 0.0000, 0.5946)
			H1 (0.3042, 0.2543, 0.2730)
			H2 (0.7690, 0.2525, 0.5909)
			H3 (0.5128, 0.0000, 0.0326)
			H4 (0.1348, 0.0000, 0.7145)
			H5 (0.6973, 0.5000, 0.1377)
			H6 (0.0394, 0.5000, 0.4112)
			H7 (-0.0105, 0.5000, 0.8980)
*Cmcm*	450	a = 2.469 b = 8.044 c = 3.414	Ca1 (0.0000, 0.3863, 0.7500)
		$\alpha$ = 90 $\beta$ = 90 $\gamma$ =90	C1 (0.0000, 0.1042, 0.7500)
			H1 (0.0000, 0.5947, 0.7500)
			H2 (0.0000, 0.7444, 0.7500)
			H3 (0.0000, 0.2040, -0.5160)
			H4 (0.0000, 0.0000, 0.5000)

The stable structure of the *Pm* and *Cmcm* structures can be considered from a characteristic bonding through a uniform electron gas of the same density. We show here that the electron localization function (ELF)^31^ can be used to analyze the stability of high pressure structure as has been demonstrated in several materials^15,32–37^. First of all, we can observe the electrons distribution in the *Pm* structure at 50 GPa, which is plotted in the (010) plane (Fig. 2c), it showed that the distribution of electrons between the C atom and the H atom reveals significant strong bonding. The distribution of electrons between C and C is also reveals a strong bonding. On the other hand, the Ca atom is not likely to bond with the C and H atoms. Moreover, we found that the *P*m structure displayed the electron accumulated considerably around H-atom with respect to the Ca and C atoms. Next, the analysis of *Cmcm* structure at 450 GPa which is plotted in the (100) plane (Fig. 2d) shows that the plane between four neighboring C atoms exhibited the sparse distribution of electrons between the C atom and the H atom, showing a strong bonding. Also, the distribution of electrons between the H atom and the H atom showed a strong bonding while there was a slight accumulate between the Ca atoms and the H atoms.

**Figure 3:**
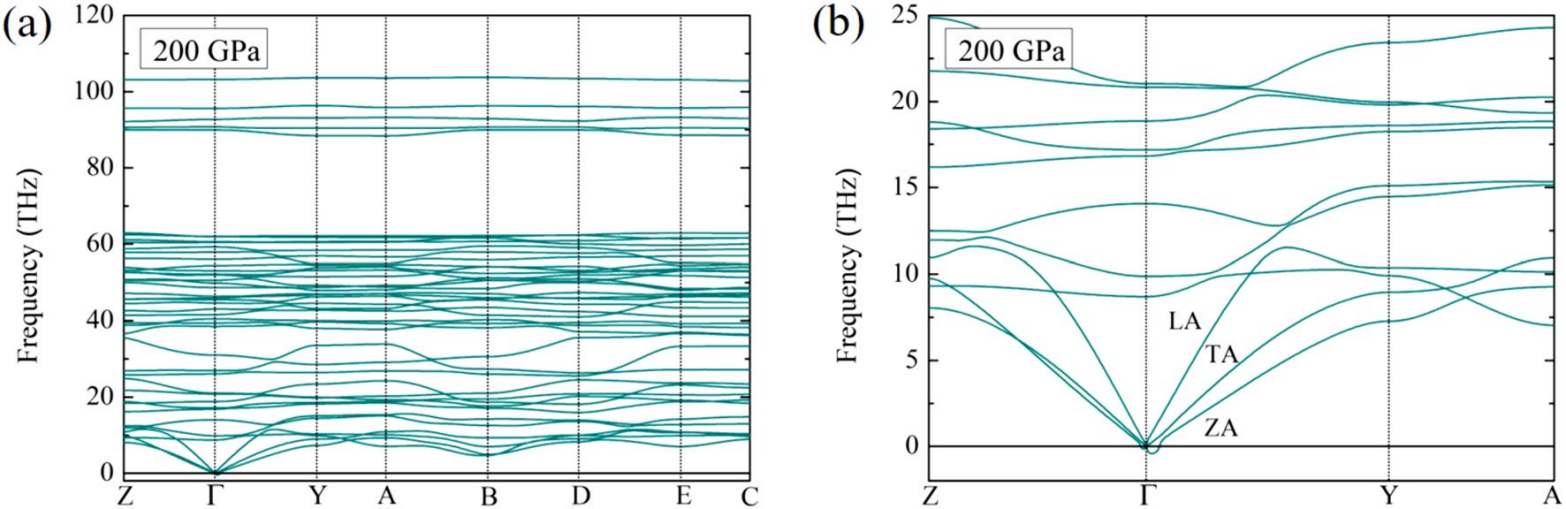
(**a**) The phonon dispersion of the *Pm* structure at a pressure of 200 GPa. (**b**) The soft-mode at the $\Gamma$–point of the *Pm* structure at a pressure of 200 GPa.

We began to consider the phonon of the *Pm* structure due to the dynamically stable structure can be used to confirm the ground-state structure. Although we found that the *Pm* structure is thermodynamically stable at 50 GPa, but this is not sufficient to a guarantee the thermodynamic stability. First, we calculated the phonon dispersions at the pressure of 50 GPa and found that the *Pm* structure is dynamically stable. Second, with the increasing a pressure up to 200 GPa, the *Pm* structure is theoretically calculated dynamical stability. It is found that there is an imaginary frequency at *Γ*-point. This evident can be clearly seen in Fig. 3a. To further our understanding, we focused the phonon dispersions at acoustic mode, as shown in Fig. 3a. Owing to existence of imaginary frequency, showing a tiny soft-mode at the $\Gamma$-point. We are focusing on the low frequency modes, which are comprise of longitudinal (L), transverse (T) and out-of-plane (Z) acoustic modes, LA, TA and ZA, respectively (Fig. 3b). We found that the soft-mode at the $\Gamma$-point occurred from ZA mode, leading to the imaginary frequency. This suggests a probable high-pressure phase above 200 GPa.

**Figure 4:**
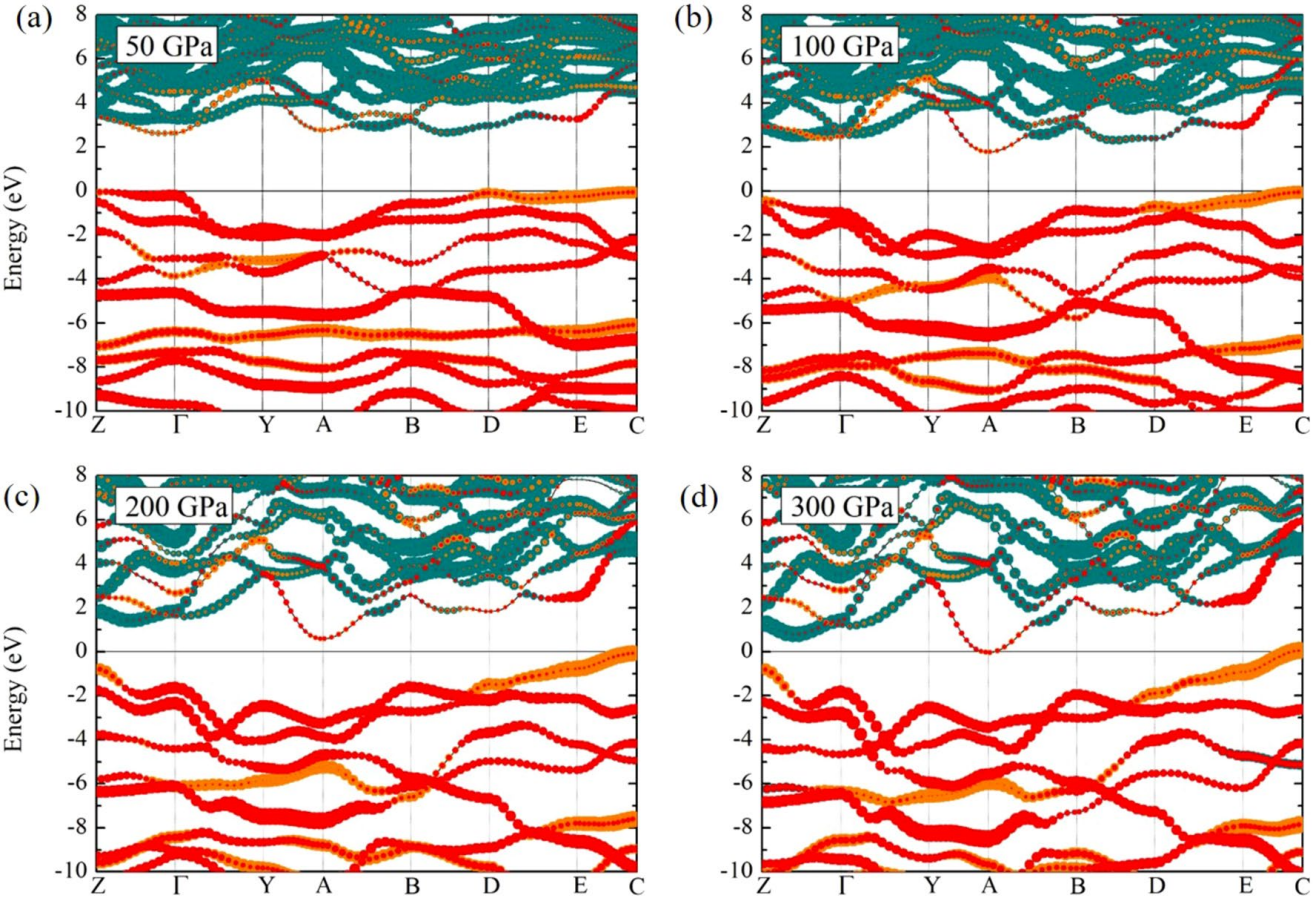
The band structure of $\hbox {CaCH}_{{5}}$ (**a**) the *Pm* structure at 50 GPa, (**b**) the *Pm* structure at 100 GPa, (**c**) the *Pm* structure at 200 GPa, and (**d**) the *Pm* structure at 300 GPa, respectively. The dark cyan, orange, and red circles represent, respectively, Ca, C, H atoms.

In order to investigate further into the physical property of this *Pm* structure, we calculated the electronic band structure as the physical property is the first and most significant advantage. As a result of this, the *Pm* structure displayed the semiconductor, it is seen from Fig. 4. To start with the energy gap at a pressure of 50 GPa, we found that the energy gap is 1.4 eV and it increased gradually by approximately 1.4 to 1.8 eV. Also, the energy gap decreased by 0.6 eV at a pressure of 200 GPa. Interestingly, we found that this trend of semiconductor towards becoming a metal at a pressure of 300 GPa, as shown in Fig. 4d.

We now discuss the electronic band structure of the *Pm* structure which is shown in Fig. 4, the band structure manifested an intrinsic semiconductor at a pressure of 50 GPa. With increasing pressure, the band structure showed the downward shift of the conduction band at the A-point at a pressure of 100 GPa. According to the band structure calculations, when greater pressure is applied, one can see that the conduction band displayed the downward shift at the A-point. Eventually, the conduction band’s downward shift at the Fermi level. As a result of this, the *Pm* structure exhibited the electronic topological transition (ETT), which referred to as the Lifshitz transition^38^. This because the Lifshitz transition is a kind of ETT, it should be mentioned that the ETT is change of the band shape under high pressure^39,40^. It is well-known that the Lifshitz transition is associated with an ETT because the ETT originates from the electronic band structure. Likewise, the topology of materials^41–45^ shows that the ETT changes through compression. Following this, we pointed out that a role of Lifshitz transition led to semiconductor to metal because the electrons occupied at the Fermi level at a pressure of 300 GPa. At this point, it should be noted that the structural phase transformation was examined by the GGA-PBE functional framework. In order to stay within the same motif, the electronic band structure calculations performed the GGA-PBE functional. It generally accepted that the GGA-PBE functional underestimated the band gaps. Therefore, we suggested that the *Pm* structure might correct the band gaps for further theory to explore the hybrid functional such as the Heyd–Scuseria–Ernzerhof (HSE) functional. Here again, as aforementioned theoretical findings, it is interesting to note the remarkable result of phonon calculations of the *Pm* structure. This because we have mentioned that it is possible for stabilization of $\hbox {CaCH}_{{5}}$ above 300 GPa. In consequence of the result of the electronic band structure, it worth noting that $\hbox {CaCH}_{{5}}$ is likely to be metallic at extremely high pressure. We thus tried to explore this possibility by investigate the electronic structure of the *Cmcm* structure.

**Figure 5:**
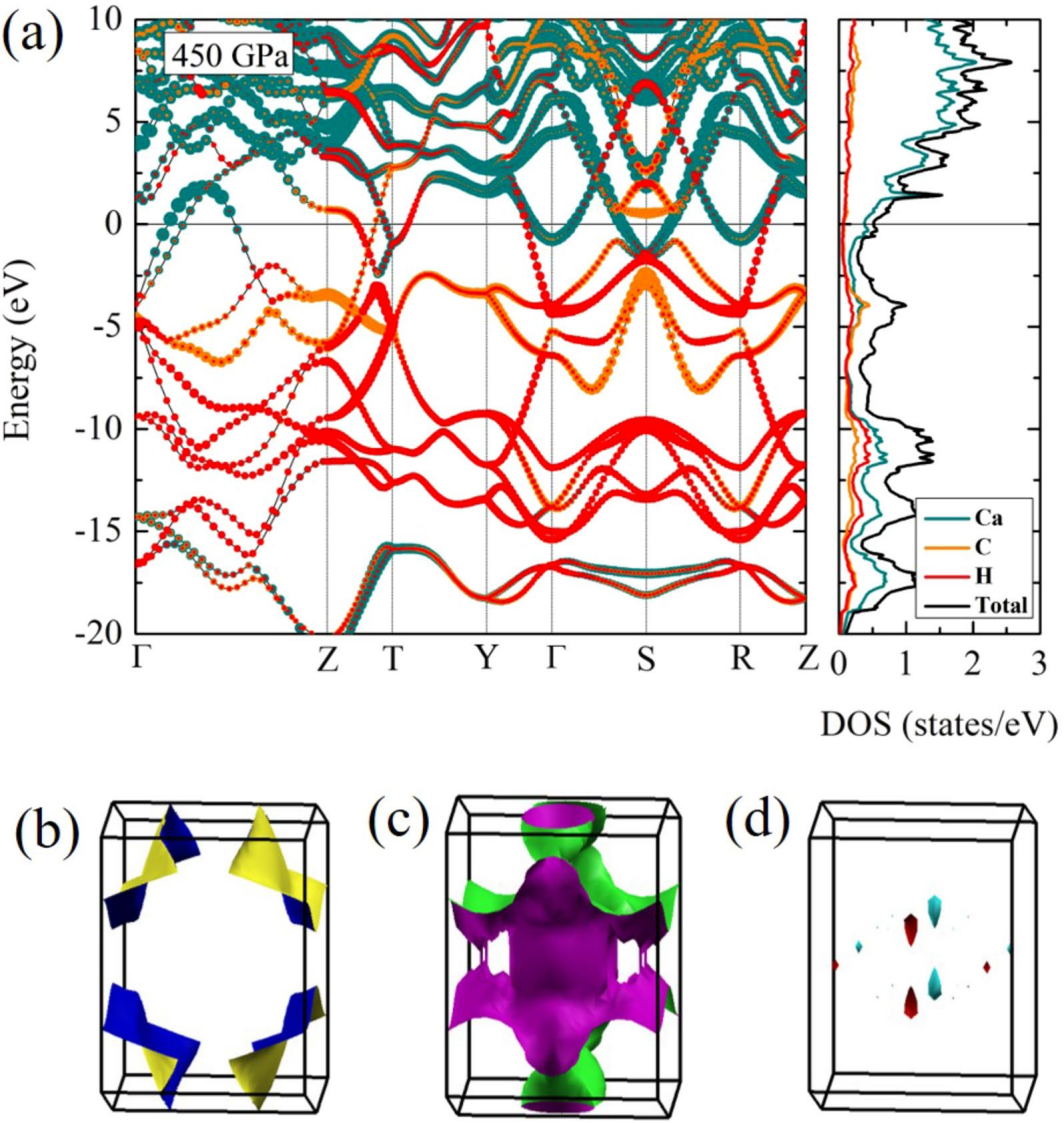
(**a**) The band structure of the *Cmcm* structure, where the dark cyan, orange, and red circles represent, respectively, Ca, C, H atoms, and density of states the *Cmcm* structure, where the dark cyan, orange, and red lines represent, respectively, Ca, C, H atoms, at 450 GPa. (**b**–**d**) the Fermi surface of the *Cmcm* structure at 450 GPa (drawn by XCrySDen program (ver. 1.5.60)^57^ (URL http://www.xcrysden.org/Download.html#_toc_1)).

To describe the possibility of superconductivity, we now fully investigate the electronic band structure of the *Cmcm* structure. This is can be observed in Fig. 5a, where we have shown the electronic band structures at a pressure of 450 GPa. It can see that the *Cmcm* structure is metal because the valence and conduction bands crossed around the Fermi level. According to the electronic band structure, it is worth noting that steep bands accommodate localized electrons around the Fermi surface. This indicates that the steep band is achieved even for conventional superconductors. Following this, the steep band which has high velocity electrons, according to conventional superconductivity are responsible for electron-electron correlations as forming Cooper pairs^46,47^. Figure  5a shows the illustration of the steep/flat band, it should be mentioned that there remains the of the steep bands interact with the flat bands at S-point. Consequently, it can see that the steep band is extremely large in comparison to the flat band. Therefore, studying the Fermi surface plays a critical role for superconductivity^41,42^. Regarding the Fermi surface, the characteristics of the Fermi surface have been successfully determined for several materials^15,37,48,49^. Therefore, in this work, the $\hbox {CaCH}_{{5}}$ and its Fermi surface are theoretically studied at a pressure of 450 GPa. As result of this, these Fermi surfaces exhibited Fermi surface nesting as can be seen in Figs. 5b, 5c, and 5d, respectively. This in turn implies that a trend of $\hbox {CaCH}_{{5}}$ towards becoming the superconductor. In addition, the *Cmcm* phase is calculated at the pressure of 450 GPa and found that there is the Fermi surface nesting. To the best of our knowledge, the Fermi surface nesting play an important role for an effective in enhancing the EPC^50,51^. Following this, it is therefore interesting to further suggest as the Fermi surface nesting can point out that it can be associated with the phase transition^50^, arising particularly from the driving forces. For this particular case, when the *Cmcm* structure is extremely compressed. $\hbox {CaCH}_{{5}}$ is likely to be attained theoretically for an occurrence of novel structure. This in turn implies that the structure of $\hbox {CaCH}_{{5}}$ is possible to transition from the *Cmcm* structure to novel phase, by adopting compression above 500 GPa.

**Figure 6:**
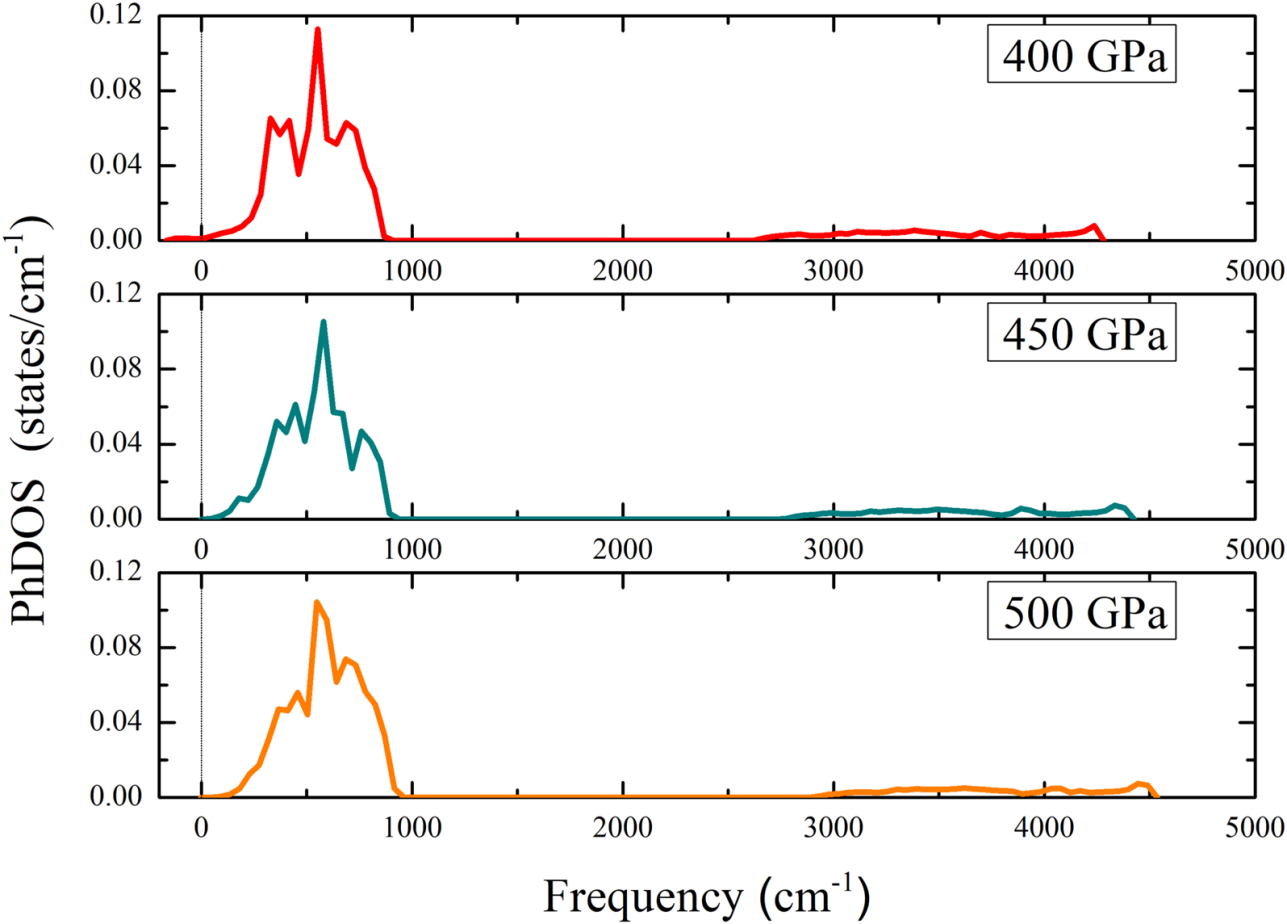
The phonon density of states of the *Cmcm* structure at a pressure of 450 GPa, 450 GPa, and 500 GPa.

**Figure 7:**
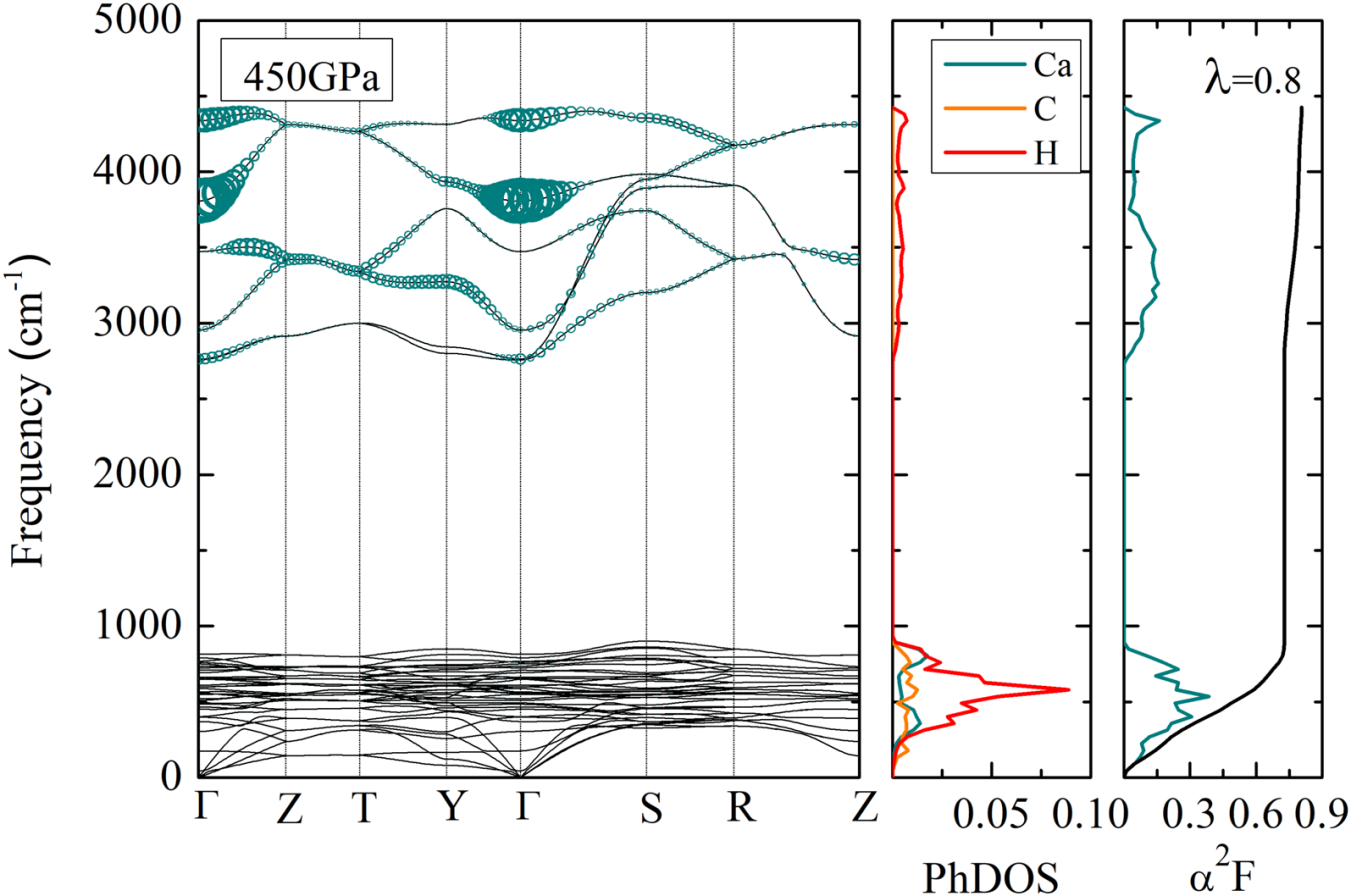
The phonon dispersion of the *Cmcm* structure, phonon density of states, the Eliashberg spectral function of the *Cmcm*, and the integration of the lambda of the *Cmcm* at 450 GPa. Magnitude of partial EPC parameter represents by the circle on the phonon dispersion plot.

Regarding the dynamically stable structure, we have completed the phonon calculations by the linear response method. The remarkable results, the phonon density of state (phDOS), showed that the *Cmcm* structure is the dynamically unstable structure at a pressure of 400 GPa. As a possible cause of this, one might think of the quantum effects. This because the quantum effects are important for the structural stabilities of materials with high EPC constants^17,37,52^ such as $\hbox {S}_{0.5}\hbox {P}_{0.5}\hbox {H}_{{3}}$^17^, $\hbox {BC}_{{7}}$^37^, and $\hbox {LaH}_{{10}}$^52^, it is possible that EPC can destabilize quantum effects. For this particular case, it should be mentioned that the *Cmcm* structure is a dynamically unstable structure at the high-pressure limit (400 GPa). This puts in question the mechanism of superconductivity, thus deserving further clarification. By taking into consideration the fact that a $\hbox {T}_{{c}}$ is generally required to achieve the EPC. Herein, it seems that quantum effects are one of the influencing mechanisms of superconductivity, indicating that the *Cmcm* structure exhibited anharmonic phonons which explain the Hessian of the quantum energy^52^ At this point, we suggested that its $\hbox {T}_{{c}}$ can be calculated from the anharmonic quantum calculations within the stochastic self-consistent harmonic approximation (SSCHA)^53,54^ This paves the way for further studies on the effect of pressure. On compression, one can see that the phDOS displayed that the *Cmcm* structure is dynamically stable, starting from a pressure of 450 GPa, as shown in Fig. 6. On further compression to 500 GPa, it also showed that the *Cmcm* structure is dynamically stable. The results can point out that it is worth predicting the value of $\hbox {T}_{{c}}$.

Therefore, it is interesting to calculate the $\hbox {T}_{{c}}$ of the *Cmcm* structure at a pressure of 450 GPa, as shown in Fig. 7; we computed the EPC by the linear response method because it successfully demonstrated an Eliashberg spectral function. In order to consider the superconducting properties, it is well-known that the GGA-PBE functional is sufficient for achieving the $\hbox {T}_{{c}}$. Firstly, the result of Eliashberg spectral function of the *Cmcm* structure is contributed mainly by approximately 0 cm$^{-1}$ to 900 cm$^{-1}$ and by approximately 2800 cm$^{-1}$ to 4500 cm$^{-1}$. We found that the integration of lambda increased dramatically by approximately 0 to 900 cm$^{-1}$. After that, it can be seen that there was a constant remain to around 2800 cm$^{-1}$; besides, the integration of lambda increased steadily to the highest frequency, and we found that it reached a peak of 0.8. At this stage, we will continue to calculate the value of $\hbox {T}_{{c}}$. The remarkable result shown that there is the $\hbox {T}_{{c}}$ is 50 K and the $\omega _{log}$ is 1059 K which used $\mu ^{*}$= 0.10. Moreover, we calculated the integration of lambda is 0.64 is calculated at a pressure of 500 GPa and found that the $\hbox {T}_{{c}}$ is 46 K and the $\omega _{log}$ is 1653 K. Following this, the nature of the $\hbox {T}_{{c}}$ decreased with increasing pressure. For example, several materials observed successfully in the $\hbox {T}_{{c}}$^14,15,18,34,55^ such as $\hbox {CeH}_{{10}}$^15^, and Hf$H_{6}$^18^. To further analysis the phonon dispersion, it is interesting to note that a magnitude of partial electron phonon coupling parameter represents by the circle on the phonon dispersion plot. Herein, we would like to elucidate the mechanism of the $\hbox {T}_{{c}}$ at a pressure of 450 GPa. As a result of this, we found that optical phonon mode mostly dominated by hydrogen. In addition, the magnitude of partial electron phonon coupling parameter is remarkably large for those $\Gamma$-point. Consequently, it might be expected that the $\hbox {T}_{{c}}$ of $\hbox {CaCH}_{{5}}$ reaches about 50 K at a pressure of 450 GPa. Additionally, their $\hbox {T}_{{c}}$ decreased with the increasing pressure. As a possible cause of this, one might think of the integration of lambda decreased with increasing pressure. Moreover, It is interesting to compare with $\hbox {CaBH}_{{5}}$^19^. We found that the superconducting phase in $\hbox {CaBH}_{{5}}$ is predicted to be 300 GPa but the superconducting phase in $\hbox {CaCH}_{{5}}$ is predicted above 450 GPa. Thus, they can not be compare directly under the same pressure. However, we suggested that for both $\hbox {CaBH}_{{5}}$ and $\hbox {CaCH}_{{5}}$ can be considered from B and C through Pauling electronegativity. For instance, the case of $\hbox {HfH}_{{10}}$ and $\hbox {ZrH}_{{10}}$ manifested an important role of Pauling electronegativity^16^. As a result, they described the elements of Hf and Zr, showing a similar Pauling electronegativity. For our work, Pauling electronegativity of C (2.55) has moderately larger than Pauling electronegativity of B (2.04). As a possible cause of this, one might think of Pauling electronegativity is a key factor for the value of $\hbox {T}_{{c}}$, leading to the potential to enhance $\hbox {T}_{{c}}$ in the Ca–C–H system.

## Conclusion

In summary, we explore the high-pressure phase of $\hbox {CaCH}_{{5}}$ by first-principles evolutionary algorithm, based on density functional theory. Within structural stability, we have shown that there are two novel structures which likely to be stable structures. They are *Pm* structure and the *Cmcm* structure. Firstly, the perspective of theoretical inspection reveals that the *Pm* structure is the semiconducting phase. Secondly, $\hbox {CaCH}_{{5}}$ shows a possible way for achieving stabilization of superconducting phase via the electronic topological transitions of the *Pm* structure. Along with, the *Pm* structure is likely to be a metallic phase at a pressure of 300 GPa. Without any doubt, we demonstrate that the *Cmcm* structure is the most stable structure. Subsequently, the *Cmcm* structure is the superconducting phase which $\hbox {T}_{{c}}$ is estimated to be 50 K at a pressure of 450 GPa. The new structure suggested here establishes a further criteria for superconductivity in the ternary metal pentahydride compounds.

## Supplementary Information

Supplementary Information.

## Data Availability

The data that support the findings of this study are available from the corresponding author upon reasonable request.
