# Supplementary material for: Stabilizing superconductivity of ternary metal pentahydride CaCH5 via electronic topological transitions under high pressure from first principles evolutionary algorithm
Source: Sci Rep. 2022 Apr 25;12:6700. doi: 10.1038/s41598-022-10249-1 (PMC9039074; doi:10.1038/s41598-022-10249-1)
Supplement: Supplementary file 1 — Supplementary Information. [file 41598_2022_10249_MOESM1_ESM.docx]

Supplemental Materials

of

Stabilizing superconductivity of ternary metal pentahydride CaCH5 via electronic topological transitions under high pressure from first principles evolutionary algorithm

Prutthipong Tsuppayakorn-aek^1,2^, Nakorn Phaisangittisakul^1,2^, Rajeev Ahuja^3,4^, and Thiti Bovornratanaraks^1,2,**^

^1^Extreme Condition Physics Research Laboratory and Center of Excellence in Physics of Energy Materials (CE:PEM), Department of Physics, Faculty of Science, Chulalongkorn University, Bangkok 10330, Thailand

^2^Thailand Centre of Excellence in Physics, Ministry of Higher Education, Science, Research and Innovation, 328 SiAyutthaya Road, Bangkok 10400, Thailand

^3^Condensed Matter Theory Group, Department of Physics and Materials Science, Uppsala University, Box 530,SE-751 21, Uppsala, Sweden

^4^Department of Physics, Indian Institute of Technology (IIT) Ropar, Rupnagar 140001, Punjab, India

Corresponding author [**thiti.b@chula.ac.th](mailto:**thiti.b@chula.ac.th)

**Computational details**

The formation enthalpy calculation was calculated using the first-principles calculations, based on the density functional theory, as implemented in the VASP code [1] A plane-wave basis set up to cutoff energy of 700 eV and an initial Brillouin-zone (BZ) sampling grid of spacing 2π×0.02 Å^−1^ were used for this calculation. All structures were fully relaxed using the generalized gradient approximation of the Perdew–Burke–Ernzerhof (GGA-PBE) functional. [2]

**Result and discussion**

Overall, the solution of the convex hulls calculations manifested that the formation enthalpies, as shown in Figure S1. The theoretical basis is an formation enthalpy, by adopting the convex hulls, where enthalpy formation as defined

$${\Delta H}^{f}=\frac{H_{{CaCH}_{5}}-\left( N_{CaC}H_{CaC}+N_{H}H_{H} \right)}{\left( N_{CaC}+N_{H} \right)}$$

Where *N* and *H* are the number of atoms and enthalpy per atom of element, respectively. Following this, a negative enthalpy indicates that a structure is stable with respect to decomposition into its elements. We have shown that the stability of CaCH_5_ up to at least 500 GPa as. As a result of this, it displayed that the relative enthalpy of CaCH_5_ is negative enthalpy with respect the phase decomposition into the CaC and H phases from 100 GPa to 500 GPa. This is in turn implies that CaCH_5_ against the phase decomposition into the CaC and H phases. For this particular case, we consider the formation enthalpy of CaCH_5_ respect to calcium carbide and hydrogen, [3,4] Moreover, a synthesis of CaCH_5_ can consider formation-route types. For ternary metal hydrides can be summarized: A + B + C → D; A+ B → D; A + B → D + E, etc. For the case of CaCH_5_, the A + B → D type formation route is mainly concerned. Thus, it could be synthetized by CaC+5H→ CaCH_5_. For these reason, CaCH_5_ can be synthesized thought the theoretical basis.


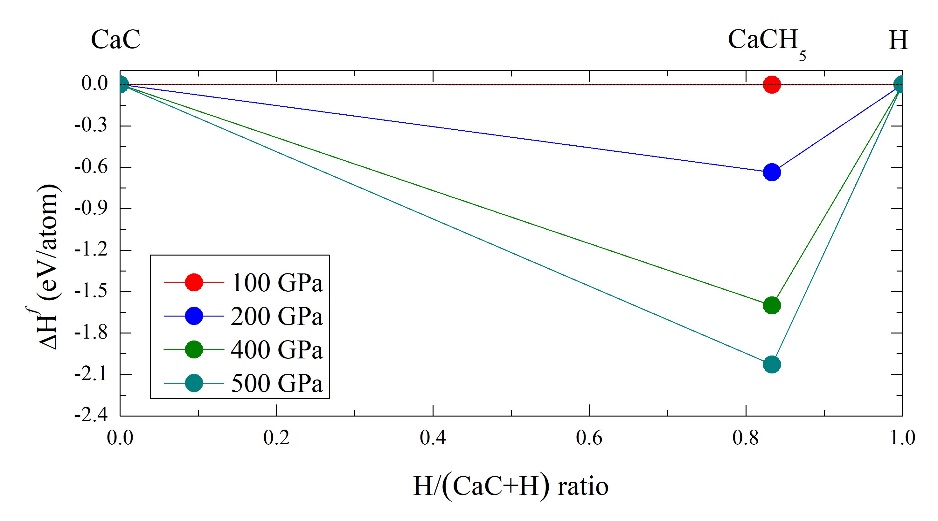


Figure S1: The convex hull diagram is calculated by the enthalpy formation.

**References**

[1] Kresse, G. & Furthmüller, J. Efficient iterative schemes for ab initio total-energy calculations using a plane-wave basis set. Phys. Rev. B 54, 11169–11186 (1996).

[2] Perdew, J. P., Burke, K. & Ernzerhof, M. Generalized gradient approximation made simple. Phys. Rev. Lett.77, 3865–3868 (1996).

[3] L. Beldi et al., Comput. Condens. Matter, 17, 00336 (2018)

[4] C. J. Pickard and R. J. Needs, Nat. Phys, 3, 473 (2007)
